# Differential expression of microRNA in serum fractions and association of Argonaute 1 microRNAs with heart failure

**DOI:** 10.1111/jcmm.15306

**Published:** 2020-05-13

**Authors:** Eti Meiri, Natalia Volinsky, Nir Dromi, Sharon Kredo‐Russo, Hila Benjamin, Sarit Tabak, Hagai Marmor, Maria Motin, Danit Lebanony, Gila Lithwick‐Yanai, Etti Kadosh, Carol Kreader, Liza Grosman‐Rimon, Offer Amir

**Affiliations:** ^1^ Rosetta Genomics Ltd Rehovot Israel; ^2^ Division of Cardiovascular Medicine Baruch Padeh Medical Center Poriya Israel; ^3^ The Azrieli Faculty of Medicine in the Galilee Bar‐Ilan University Safed Israel; ^4^ MilliporeSigma Merck KGaA Darmstadt Germany

**Keywords:** argonaute, heart failure, microRNA

## Abstract

The serum or plasma microRNA (miRNA) molecules have been suggested as diagnostic and prognostic biomarkers, in various pathological conditions. However, these molecules are also found in different serum fractions, such as exosomes and Argonaute (Ago) protein complexes. Ago1 is the predominant Ago protein expressed in heart tissue. The objective of the study was to examine the hypothesis that Ago1‐associated miRNAs may be more relevant to cardiac disease and heart failure compared with the serum. In total, 84 miRNA molecules were screened for their expression in the whole serum, exosomes and Ago1, and Ago2 complexes. Ago1‐bound miR‐222‐3p, miR‐497‐5p and miR‐21‐5p were significantly higher, and let‐7a‐5p was significantly lower in HF patients compared with healthy controls, whereas no such difference was observed for those markers in the serum samples among the groups. A combination of these 4 miRNAs into an Ago1‐HF score provided a ROC curve with an AUC of 1, demonstrating clear discrimination between heart failure patients and healthy individuals. Ago1 fraction might be a better and more specific platform for identifying HF‐related miRNAs compared with the whole serum.

## 1 INTRODUCTION

microRNAs (miRNAs) are small, non‐coding RNA molecules consisting of 19‐24 bases in their mature form. miRNA usually bind to the 3’‐untranslated region of messenger RNA (mRNA) and down‐regulate protein expression by one of two independent mechanisms: targeting mRNA to degradation or preventing its translation into protein. The latter mechanism is most common in animal tissues. At least 60% of all human genes can be potentially regulated by miRNA,^1^ with each mRNA targeted by several miRNA molecules, whereas each miRNA molecule can repress expression of multiple genes to different degrees, thus creating a complicated network of miRNA‐mRNA interactions.^2^

miRNA molecules are transcribed as primary, non‐coding transcripts (pri‐miRNA), containing a stem‐loop structure. pri‐miRNA is processed by nuclease DROSHA into miRNA precursors (pre‐miRNA), which is further processed by RNase III‐like enzyme DICER into a mature miRNA duplex.^1^, ^3^ miRNA duplex is loaded into the Argonaute (Ago) protein found in the RNA‐induced Silencing Complex (RISC). The passenger strand of miRNA duplex is removed, whereas the guide strand is used for recognition of target mRNA. miRNA within the RISC complex binds target mRNA at a rate of up to 250 times faster than in a protein‐free form.^4^ Thus, Ago found within the RISC complex facilitates and enables mRNA targeting and translational repression. In humans, the Ago family consists of 4 (1‐4) proteins. Ago1 and Ago2 are the two most commonly expressed Ago proteins in humans; however, no preference in miRNA molecules binding between Ago1 and Ago2 was found.^5^, ^6^ At the same time, Ago proteins demonstrate tissue‐specific expression^7^, ^8^; for instance, Ago1 is a predominant protein in adult human kidneys and the heart.^7^ Unlike in cell lysates, Ago1‐ and Ago2‐assosiated miRNA detected in plasma do not correlate with each other and with miRNAs found in blood cells, suggesting their source is from different tissues.^5^

Whether there are differences in the expression of miRNAs in different serum fractions in healthy and heart failure (HF) patients is not clear. HF is a terminal stage of most cardiovascular diseases. The lifetime risk of developing HF is 1 in 5.^9^ Although several biomarkers, such as N‐terminal pro‐Brain Natriuretic Peptide (NT‐proBNP) are widely used for diagnostic purposes of HF, miRNAs secreted by a failing heart can be used for diagnostic or prognostic purposes.^10^, ^11^, ^12^, ^13^, ^14^ Identification of miRNAs associated with the condition might contribute to our current knowledge regarding the HF pathogenesis and improve patients' treatment in the future. Total serum is commonly used to identify miRNAs associated with cardiovascular diseases,^12^, ^13^, ^14^ though some of these miRNAs may potentially be missed due to them being masked by the same miRNA molecules found in serum that may originate from another source, such as blood cells.

In the current study, we aimed to explore miRNAs found in different serum fractions: whole serum, exosomes, and Ago1 and Ago2 protein complexes in healthy and HF patients, and compare their potential differential expression between serum fractions. We hypothesized that miRNA sampling from serum fractions may enable us to overcome the limitation of miRNA masking and promote better identification of miRNAs associated with particular pathological conditions. Specifically, given that Ago1 is the predominant Ago protein in heart tissue, we hypothesized that measuring miRNA expression in Ago1 fraction may enable more accurate identification of miRNAs that are associated with HF pathology.

## 2 METHODS

### 2.1 Cohort characteristics

In total, 18 participants were included in the study. Eight patients with chronic systolic HF were recruited to the study. All patients [left ventricular ejection fraction (LVEF) <40%] were treated for at least 3 months according to the ACC/AHA guidelines; guided directed medical therapy was applied as follow: Angiotensin‐converting enzyme inhibitor/Angiotensin II receptor blocker was applied in all patients and Aldosterone antagonists in 4 (50%) patients. All patients were stage C and clinically stable as judged by the treating HF‐specialized cardiologist on the day of recruitment. Ischemic aetiology was present in 6 (75%) patients and New York Heart Association class 1‐3 in all eight patients.

In addition, a control group consisting of 10 volunteers who were matched by age, gender and ethnicity to the HF group were recruited. The inclusion criteria for the control group were as follows: absence of any known coronary, valvular, or myocardial diseases. Comorbidities for coronary artery disease such as diabetes mellitus, hypertension, hyperlipidaemia and smoking did not preclude recruitment. Exclusion criteria for all participants were as follows: pregnancy, dialysis and known or treated malignancies. Clinical and demographic data were collected for the HF and control groups. In addition, echocardiographic, electrocardiographic and baseline laboratory data were recorded for the HF group.

The study was approved by the Institution Review Board of the Lady Davis Carmel Medical Center and research was carried out according to the World Medical Association Declaration of Helsinki.^15^ Informed consent was obtained from all participants prior to their participation in the study.

For the calibration steps, healthy volunteers’ fractions, different from the tested cohort, were used and tested for the following: exosomes, Ago2 and Ago1, whole serum, whole plasma, white blood cells, platelets and red blood cells.

### 2.2 Serum isolation and storage

An 8 mL aliquot of blood was collected from each participant in serum collection tubes (Greiner Bio‐one, VACUETTE^®^ Serum Tubes, 455071). The whole blood was allowed to stand for ~1 hour at room temperature before being centrifuged at 1800 *g* for 10 minutes at room temperature. The resultant serum was aliquoted into Eppendorf tubes and stored at –80°C.

### 2.3 White blood cell (WBC), platelets and red blood cell (RBC) isolation and storage

WBC fractions were isolated from 8 mL of blood that was collected into CPT collection tubes (BD Vacutainer CPT tubes, 362761, Becton Dickinson and Company), according to the manufacturer's instructions. Isolation of platelet samples was performed as described previously.^16^ RBCs were isolated from 8 mL of blood that was collected into plasma collection tubes (Greiner Bio‐one, VACUETTE^®^ Plasma Tubes 455036). Briefly, the whole blood was allowed to stand for ~1 hour at room temperature before being centrifuged at 3000 *g* for 15 minutes at room temperature. The RBC pellets were stored at –80°C after adding 1 mL mirVana Lysis/Binding Buffer to the cell pellets.

### 2.4 Exosome separation

Exosomal separation from a 0.5 mL serum was performed using the Exoquick kit (ExoQuickTM Exosome Precipitation Solution, EXOQ20A‐1, SBI) according to the manufacturer's instructions.

### 2.5 Ago1/Ago2 RNA immunoprecipitation (RIP) for calibration steps

Immunoprecipitation of miRNA was performed using monoclonal anti‐Ago1 (clone 4B8, SAB4200084, Sigma) and anti‐Ago2 (clone 11A9, SAB4200085, Sigma) produced in rat. Anti‐rat IgG (I4131, Sigma) was used in controls. Antibodies were treated with Pierce's EZ‐Link Sulfo‐NHS‐LC‐LC‐Biotin (#21338) to covalently tag the antibodies with biotin. To form AGO/anti‐AGO complexes, 100 μL of Streptavidin Mag Sepharose beads (GE28‐9857‐99) was combined with 12.5 μg of antibody and incubated for 30 minutes at room temperature, then washed twice with 1 mL of IP Lysis/Wash buffer. The mixture was combined with blood serum (1 mL per reaction), 3% IGEPAL^®^ CA‐630 (I8896, Sigma, final concentration), Protease Inhibitor Cocktail (PIC, P8340, Sigma) and RNasine (Takara, 2313A, 0.5 U/mL final concentration) and incubated for 1 hour at room temperature. Isolation of miRNA from Sepharose beads was performed using the miRNeasy kit (217004, Qiagen), after adding 200 μL of QIAzol Lysis Reagent.

### 2.6 Ago1/Ago2 RNA immunoprecipitation (RIP) for heart failure and control groups

Immunoprecipitation of miRNA was performed similarly to the calibration step, with some modifications. To form anti‐AGO beads, 100 μL of Streptavidin Mag Sepharose beads (GE28‐9857‐99) was combined with 2.5 μg of bridging antibody, Goat Anti‐Rat IgG H&L (Biotin, AB‐ab207997, Abcam) and incubated 0.5 hours at room temperature, followed by two washes with 1 mL of IP Lysis/Wash buffer. Next, 12.5 μg of antibodies: anti‐Ago1, anti‐Ago2 or anti‐rat IgG incubated for 30 minutes at room temperature with the coated beads, followed by two washes with 1 mL of IP Lysis/Wash buffer. The mixture was combined with blood serum (1 mL per reaction), IGEPAL, PIC, P8340, RNasine and incubated for 1 hour at room temperature. Isolation of miRNA from Sepharose beads was performed using a miRNeasy kit (217004, Qiagen).

### 2.7 RNA extraction

#### 2.7.1 Serum, ago RIP and exosome

RNA was extracted from a 200 mL aliquot of serum, from Exosome fraction, or after the RIP procedure, using the miRNeasy Serum/Plasma Kit (Qiagen, 217184), according to the manufacturer's instructions. Two synthetic RNAs (IDT) were spiked‐in as controls, one before adding the 1‐Bromo‐3‐chloropropane (BCP), and the second during RNA elution from the column with RNase‐free water.

#### 2.7.2 RBC, WBC and platelets

Pellets were stored at −80°C after adding 1ml mirVana Lysis/Binding Buffer to the platelets. Total RNA, including miRNA, was extracted according to the mirVana miRNA Isolation Kit manufacturer recommendations (Life Technologies, AM 1560).

#### 2.7.3 Quantitative reverse transcription—polymerase chain reaction (qRT–PCR)

qRT–PCR was performed to measure miRNA levels as described previously.^17^ Half of the extracted RNA was introduced into the poly(A) and cDNA synthesis procedure (total volume of 20 mL) with poly(A) polymerase (NEB‐M0276L) and SuperScript II reverse transcriptase (Invitrogen) for 1 hour at 37°C. The cDNA was diluted 1:20 for each PCR. The cDNA was amplified by real‐time PCR. The PCR contained a miRNA‐specific forward primer, a TaqMan probe complementary to the 3′ end of the specific miRNA sequence, as well as part of the poly(A) adaptor sequence, and a universal reverse primer complementary to the consensus 3’ sequence of the oligo tail (dT). This method has been shown to differentiate between miRNAs with a single nucleotide mismatch.^18^ The cycle number at which the fluorescence passes the threshold [cycle threshold (C_t_)] was measured for each miRNA in each sample, as described previously.^19^ The C_t_ (Cycle Threshold) values were calculated based on output from the ABI 7500 software, namely the FAMTM and ROXTM vectors, for each qRT‐PCR curve. The calculation was performed using in‐house software, in a manner similar to the way in which the ABI 7500 software calculates C_t_s. The baseline function was calculated and subtracted from the Rn (FAMTM divided by ROXTM) vector. The C_t_ was defined as the cycle in which the delta Rn vector crosses the pre‐defined, miRNA‐specific threshold.

#### 2.7.4 Serum microRNA analysis

Our initial screening started with 84 miRNAs, which were chosen based on data from previous experiments, performed by our group and other groups.^5^, ^20^ These 84 miRNAs were measured for all calibration samples (whole sera of three healthy volunteers, exosome, RIP, RBC, WBC and platelets) and for negative controls, using qRT–PCR. Of these screen miRNAs, 47 were selected for the final experiment using the 18 selected samples from the heart failure and control cohorts.

### 2.8 Data analysis and statistics

Statistical Analysis was performed by the Matlab and MedCalc Software. References to “expression” throughout the article refer to inverted C_t_s (C_t_ values subtracted from 50). Thus, high values represent high expression. Data were normalized, as described previously.^12^ For the HF vs control experiment, data were normalized similarly, by using the mean expression of a subset of 14 miRNA, instead of all of the miRNA. The 14 miRNAs selected for normalization were not previously found to be different between HF and controls, not found to be specific to blood fractions and not found to be specific to Ago1/Ago2 fractions. The normalized set also excluded miRNAs whose median C_t_ in the samples was above 37.5.

miRNAs specific to blood fractions, including RBCs, WBCs and platelets, were selected for inclusion in the 84‐miRNA qPCR plate based on previous microarray experiments, using a microarray platform of ~2200 miRNAs (data not shown). Three blood fractions, including RBC, WBC and platelets, were extracted and profiled from a single sample and served as a control. miRNAs were selected as specific to RBC/WBC for the figures if they were in the top 80th percentile of each fraction. RBC expression was greater than two C_t_s relative to both platelets and WBC. As well, WBC expression was greater than 2 C_t_s, relative to RBC. No platelet specific miRNAs were found when using similar criteria.

**FIGURE 1:**
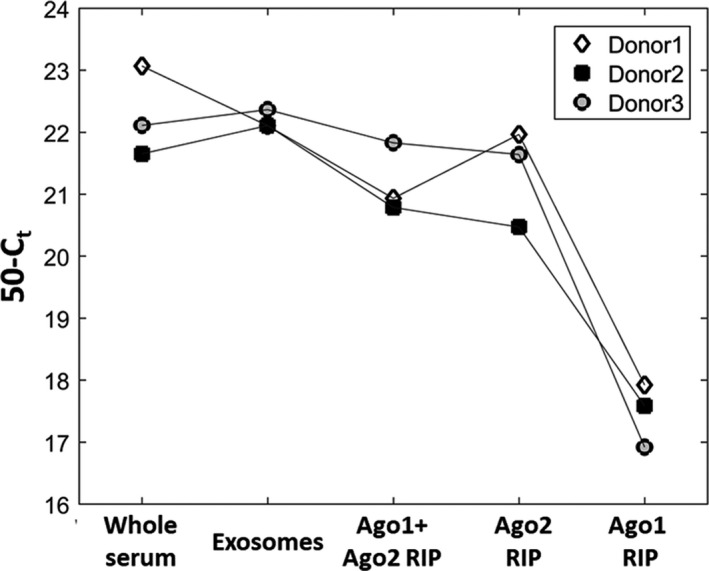
Global miRNA expression in whole serum, exosomes or in Ago1 and/or Ago2 protein complexes. Values shown are median miRNA expression in inverted C_t_ units (50‐C_t_) for each donor. For each sample type, global expression was adjusted based on the initial amount of serum used, as described in the Methods Section

Global expression for Figure 1 was defined as the median miRNA expression for a sample. Since different amounts were taken from different fractions, the global expression was adjusted based on the serum amount used for the Ago1/Ago2 RNA Immunoprecipitation: Whole serum expression was multiplied by 5, while exosome expression was multiplied by 2.

Samples were clustered using hierarchical clustering. The 40 miRNAs with the highest coefficient of variation were used for hierarchical clustering analysis. miRNAs were standardized, and 1‐Pearson correlation was used as the distance between samples.

Relative expression of miRNAs in the HF patient group was compared with the control group, using the Mann‐Whitney test. Only miRNA with a normalized median C_t_ > 12, in either Ago HF or Ago control groups, were considered for the test. Ago1‐HF score was calculated by summarizing relative expression of miR‐222‐3p, let‐7a‐5p, miR‐497‐5p, and miR‐21‐5p, either in Ago1 complexes or in whole serum. A HF score was calculated by summarizing the relative expression of miR‐22‐3p, miR‐423‐5p, miR‐320a and miR‐92b‐3p, either in Ago1 complexes or in whole serum. The ability to discriminate between the HF and control groups was characterized by the receiver operating characteristic (ROC) curve, and the area under the ROC curve (AUC) was calculated for the HF Score.

## 3 RESULTS

### 3.1 Global miRNA expression in serum fractions

First, we aimed to assess total miRNA expression in different serum fractions: whole serum, exosomes, and Ago1 and Ago2 protein complexes. Since Ago3 and Ago4 are significantly less abundant proteins than Ago1 and Ago2,^5^ they were not included in this study. Blood samples were collected from three healthy donors and serum was separated into different miRNA containing fractions. The total amount of miRNA found in exosomes was similar to the amount of miRNA found in whole serum, whereas a slight decrease in the amount of miRNA was observed in Ago1 + Ago2 RNA when using the immunoprecipitation protocol (RIP) in 2 out of 3 donors’ samples and in all 3 samples in the case of Ago2 RIP alone. The amount of miRNA associated with Ago1 is substantially lower than in any other fraction, including Ago2 complexes (Figure 1). These data suggest that the majority Ago‐associated miRNAs are found in Ago2 containing complexes, and only a small fraction of Ago‐associated miRNAs are found in Ago1 complexes. This is in line with a previous study, demonstrating Ago2 as widely expressed in hematopoietic cells, which are the major source of serum circulating miRNAs.^7^

### 3.2 Hierarchical clustering analysis

**FIGURE 2:**
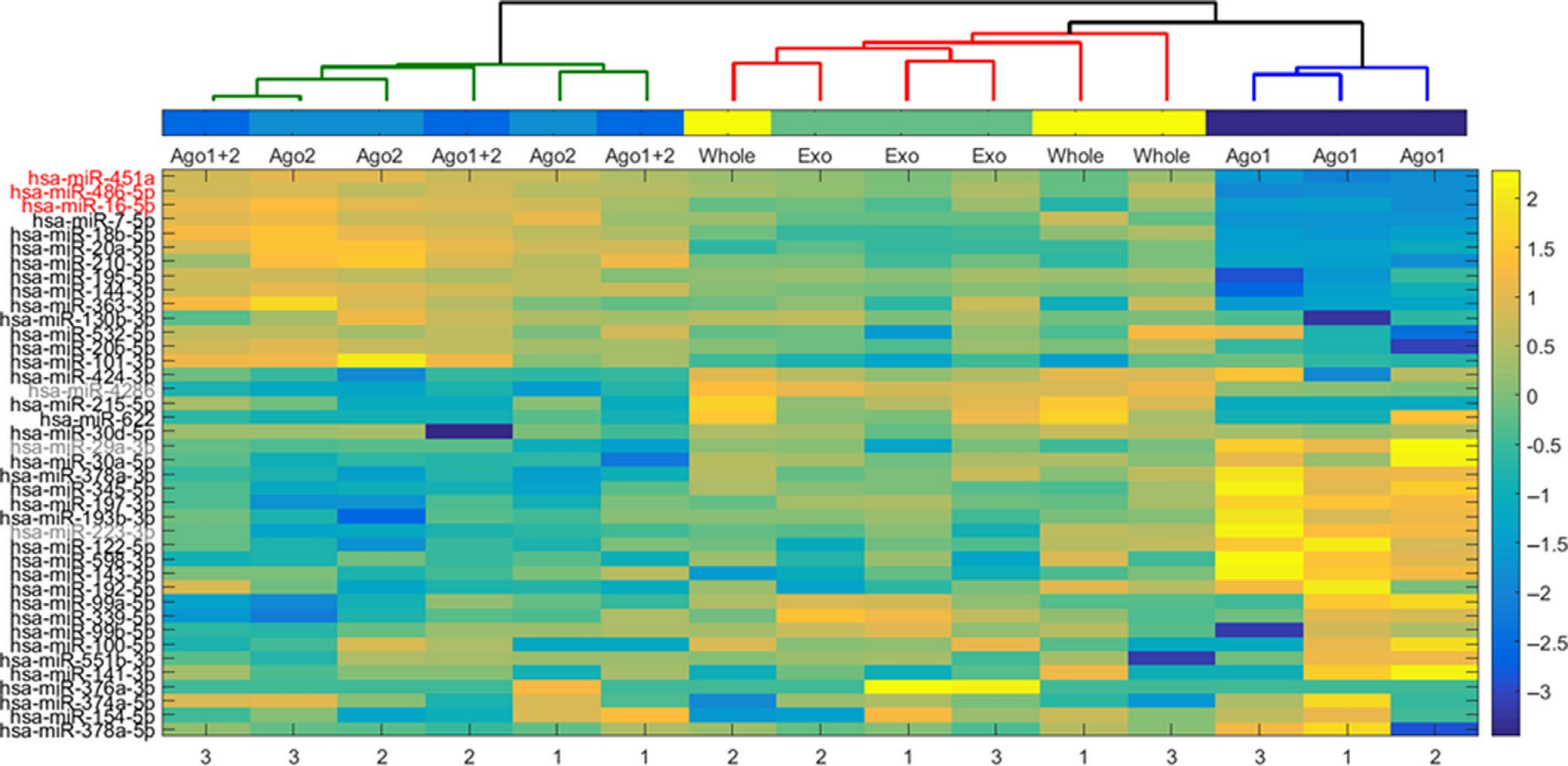
miRNA hierarchical clustering analysis. Forty out of the 84 measured miRNAs with the highest coefficient of variation were chosen for the analysis (selected miRNAs names are indicated on the Y‐axis in the left). The colored miRNAs names are RBC‐ (red) and WBC‐ (grey) associated miRNAs. The expression level of these 40 miRNAs is represented as a colored heat‐map (Y‐axis bar in the right). The correlation of miRNAs expression was used as a parameter for clustering distance. Three major clusters were formed: Ago1 + Ago2 RIP miRNAs, whole serum and exosomal miRNA and Ago1 associated miRNAs (indicated on X‐axis top). The numbers on the X‐axis in the lower bar indicate donors (n = 3)

To examine the hypothesis that Ago1 associated miRNAs are qualitatively different from miRNAs found in association with other miRNA carriers in serum, we performed a Hierarchical Clustering Analysis. Specifically, we assessed the similarity between miRNAs detected in different carriers using the hierarchical clustering analysis of the samples shown in Figure 1. Out of the 84 miRNAs measured, the 40 miRNAs with the highest coefficient of variation were chosen for the analysis. Three major clusters were formed: Ago1 + Ago2 RIP samples clustered together with Ago2 RIP samples, forming pairs of two types of samples for each donor. The second cluster contained whole serum and exosomal miRNA. Ago1 associated miRNAs formed a separate cluster, distant from Ago2 and Ago2 + Ago1 samples (Figure 2). As shown in Figure 2, there are no major differences between exosomal and whole serum miRNA. These data are consistent with a recent study by Fei Tian, where no differences were observed between miRNAs in serum and exosomes in the blood of healthy volunteers.^21^ We previously published similar results; no significant differences in miRNA expression were observed between serum and exosome‐enriched fractions, both in healthy volunteers and in heart failure patients.^12^

Co‐clustering of Ago1 + Ago2 RIP and Ago2 RIP samples can be explained by the fact that Ago1‐associated miRNAs are only a small fraction in the Ago1 + Ago2 RIP sample (Figure 1). At the same time, these data clearly demonstrate that Ago1 and Ago2 protein complexes contain different miRNA populations. Importantly, miRNAs highly expressed in erythrocytes (marked in red) are represented mainly in the Ago2 fraction and to a lesser extent, in exosomes and whole serum (Figure 2).

### 3.3 Ago1 and Ago2 specific miRNAs

**FIGURE 3:**
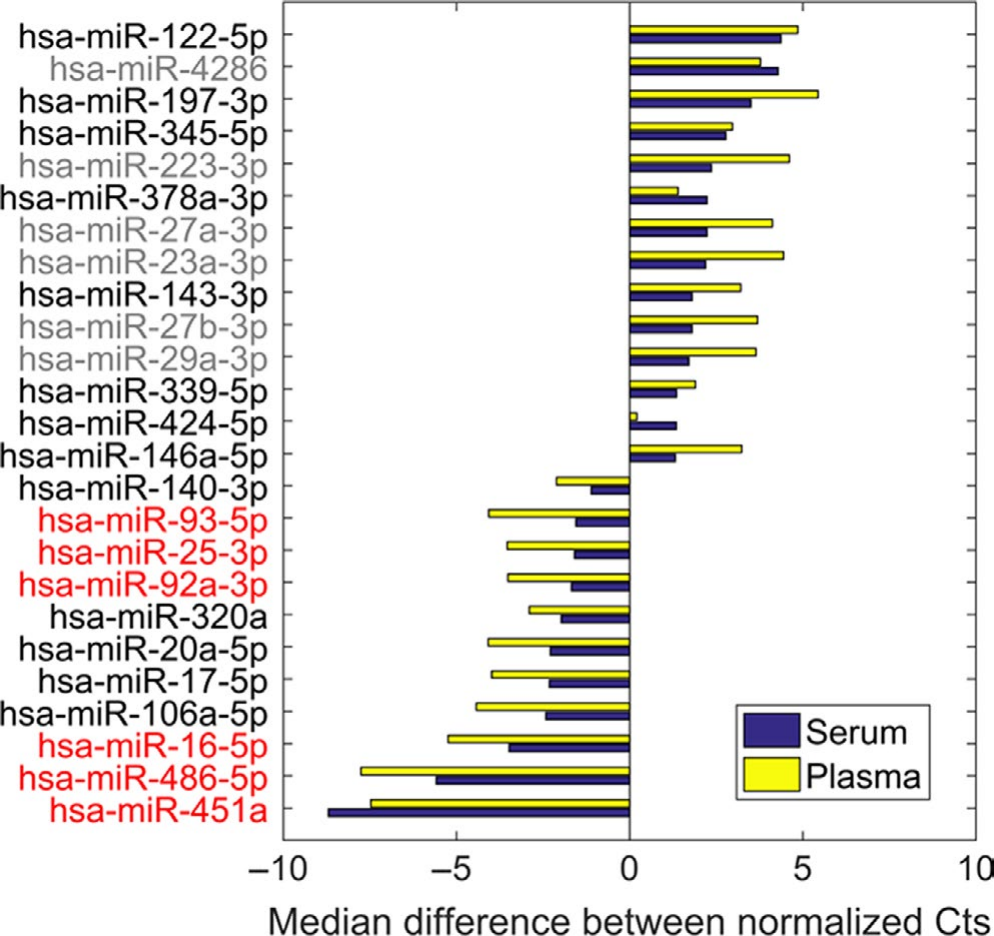
miRNAs differentially associated with Ago1 vs Ago2 proteins in serum and plasma samples. Ago1‐ and Ago2‐assosiated miRNAs detected in serum and plasma samples were selected based on the following criteria: median fold change > 2 and *P* < .05 between Ago1 and Ago2 serum samples, and C_t_ < 37.5 (for both serum and plasma samples of all 3 donors). The colored miRNAs are RBC (red) and WBC (grey) associated miRNAs. Samples of three healthy donors were used for the analysis

We identified miRNAs selectively associated with Ago1 or Ago2 in serum samples, defined as miRNAs meeting the criteria of a median fold change between Ago1 and Ago2 RIP above 2 and *P* < .05 (Figure 3). Overall, 14 miRNAs selectively associated with Ago1 and 11 miRNAs selectively associated with Ago2 were identified in serum samples. Median values of C_t_ difference for those miRNAs in serum and plasma are presented in Figure 3. Of 25 miRNAs that were significantly different between Ago1 and Ago2 serum fractions, only 3 miRNAs exceeded the threshold of *P* < .05 between Ago1 and Ago2 plasma fractions: miR‐339‐5p, miR‐378a‐3p and miR‐424‐5p. Interestingly, WBC abundant miRNAs (our unpublished data and Refs 22, 23) are mainly associated with Ago1 (eg miR‐223‐3p; marked in grey), whereas RBC specific miRNAs are present in Ago2 fractions (eg miR‐451a, miR‐486‐5p, miR‐16‐5p; marked in red) (Figure 3).

These data together with previous publications^5^, ^6^ suggest that Ago1 and Ago2 serum fractionation can potentially be used to identify tissue or cell type‐specific miRNAs, otherwise masked in whole serum by more abundant miRNAs originating from different sources.

### 3.4 HF‐related miRNAs associated with Ago1 protein

**FIGURE 4:**
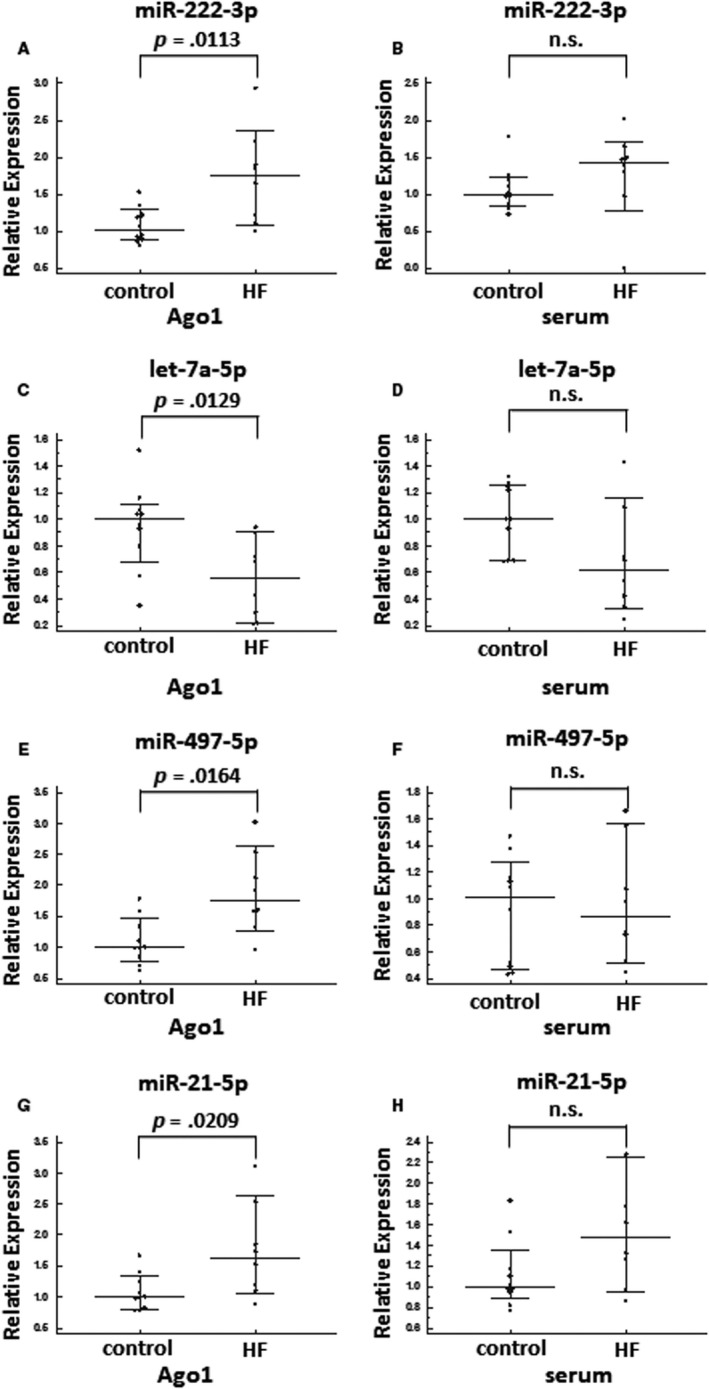
Relative expression of miR‐222‐3p (A,B), let‐7a‐5p (C,D), miR‐497‐5p (E,F) and miR‐21‐5p (G,H) in Ago1 RIP (A,C,E,G) and whole serum (B,D,F,H) of healthy individuals (n = 10) and HF patients (n = 8)

Based on the data shown in Figures 2 and 3 and on the study by Voller et al, demonstrating that Ago1 is the predominant Ago protein in the heart tissue,^7^ we tested the hypothesis that in HF patient samples (n = 8), miRNA expression will be significantly different in Ago1 RIP compared to those in healthy age and sex matching individuals (n = 10). Among 47 pre‐selected miRNAs assessed in whole serum and Ago1 RIP (Table S1), we were able to identify several up‐ or down‐regulated miRNAs in HF in Ago1 complexes. Four of the most dominant miRNAs fulfilling the criteria of fold change > 1.5 and *P* < .05 (commonly used criteria for identifying disease‐associated miRNAs^24^, ^25^, ^26^, ^27^) between HF and control groups in Ago1 samples are shown in Figure 4: miR‐222‐3p (fold change of 1.7), miR‐497‐5p (fold change of 1.8) and miR‐21‐5p (fold change of 1.6) were up‐regulated in HF patients compared with the healthy control group, whereas expression of let‐7a‐5p (fold change of −1.8) was down‐regulated. These miRNAs demonstrated similar trends in whole serum samples. However, none of them met the statistical significance criteria (Figure 4).

**FIGURE 5:**
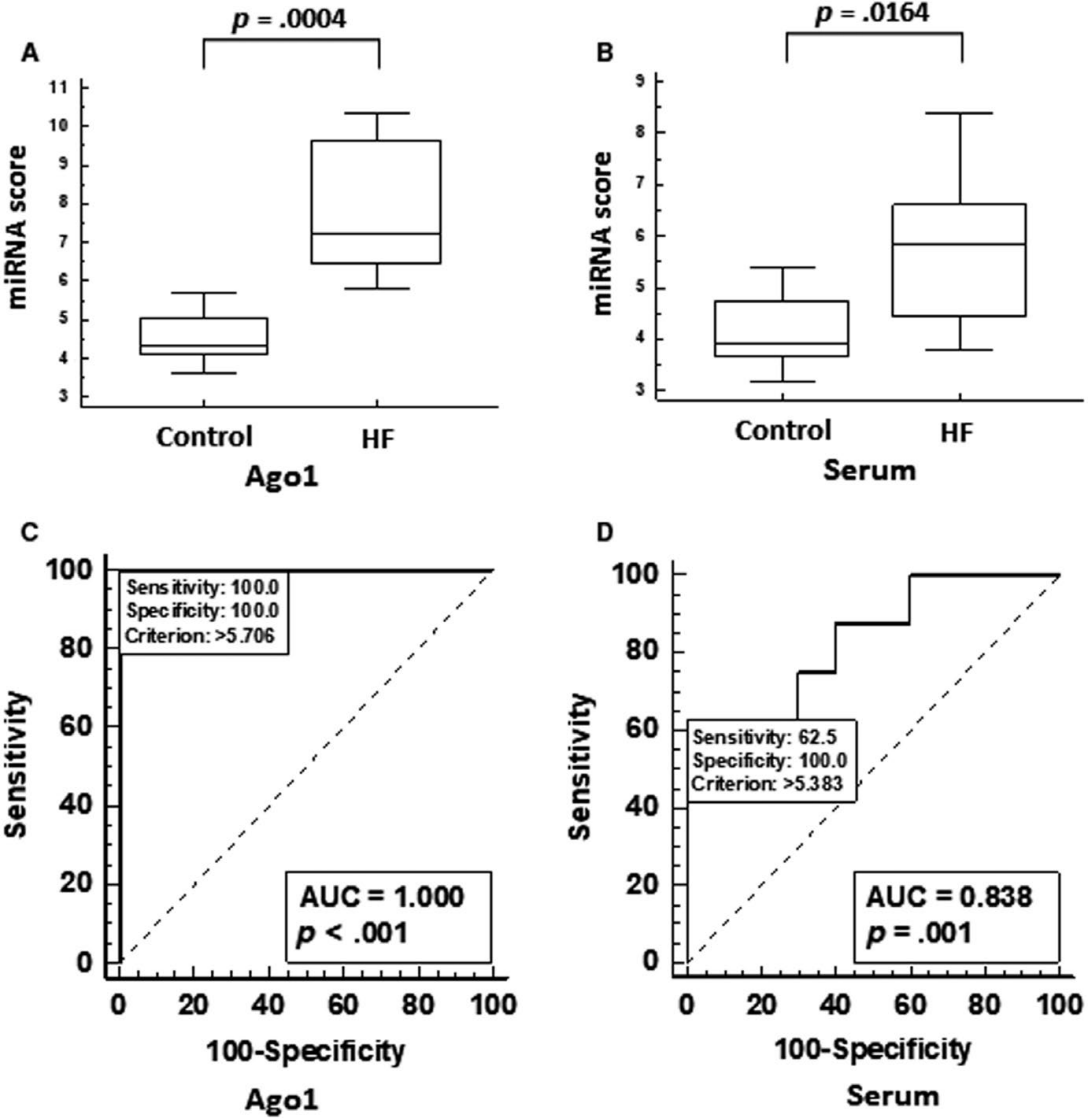
(A,B) Values of Ago1‐HF score, calculated as a sum of miR‐222‐3p, let‐7a‐5p, miR‐497‐5p and miR‐21‐5p relative expression values, in Ago1 RIP (A) and whole serum (B). (C,D) ROC curve for HF score of healthy individuals (n = 10) vs HF patients (n = 8) in Ago1 RIP (C) and whole serum (D)

To assess whether the combination of the 4 miRNAs identified in Ago1 complexes provides better distinction between HF patients and healthy individuals, compared with each individual miR, we combined relative expression levels of miR‐222‐3p, miR‐497‐5p, miR‐21‐5p and let‐7a‐5p into a single “Ago1‐HF score”. Indeed, this Ago1‐HF score provided a better distinction between the two groups compared to each individual miRNA, with a *P* value of .0004 (Figure 5A). When the same Ago1‐HF score was assessed in serum samples, the distinction between HF patients and healthy controls was much weaker, with a *P* value of .0164 (Figure 5B). In addition, to that, the ROC curve performed on the Ago1‐HF score of Ago1 samples demonstrates strong discrimination between the two groups, with an AUC of 1 and with both sensitivity and specificity of 100% (Figure 5C). Although the ROC curve of the Ago1‐HF score of serum samples demonstrated a good discrimination between the two groups of individuals with an AUC of 0.838 (Figure 5D), it was inferior to the Ago1‐HF score of Ago1 samples.

Previously we have identified miR‐22‐3p, miR‐423‐5p, miR‐320a and miR‐92b‐3p as up‐regulated in serum of HF patients, compared with healthy individuals.^12^ In this current study we asked whether Ago1 fraction will provide a better discrimination between the two groups of individuals compared with whole serum samples. A combined HF score of these four miRNAs was higher in HF patients compared to healthy individuals, both in Ago1 fraction and in whole serum, with *P* values of .0025 and .0164, respectively (Figure S1A,B). Consistent with these findings, the ROC curve of the HF‐ score of Ago1 samples demonstrated better discrimination between HF patients and healthy individuals with an AUC of 0.925 (Figure S1C) compared to the same HF score in whole serum with an AUC of 0.838 (Figure S1D). Thus, even miRNA molecules previously identified in whole serum as HF associated miRNAs, demonstrate superior results when assessed in the Ago1 fraction.

## 4 DISCUSSION

The main finding of the current study is that specific sampling of miRNAs found in the Ago1 serum fraction, enables identification of miRNAs associated with HF, which would not be otherwise identified in whole serum miRNAs expression analysis.

miRNAs secreted by the failing heart have being proposed to be useful in the diagnosis and prognosis of HF,^12^, ^13^, ^14^ with novel sensitive and high throughput methods being able to identify multiple miRNAs up‐ or down‐regulated in serum/plasma of HF patients.^28^, ^29^, ^30^ However, there could still be miRNAs secreted into the blood by a failing heart that cannot be identified in whole serum due to high levels of these miRNAs originating from different sources, such as blood cells or due to their scarcity in a sample. In addition, most miRNAs originating from RBCs, which is the main source of whole serum miRNAs, are found in Ago2 complexes.^23^ The superiority of Ago1‐HF score is supported by the knowledge that Ago1 is the predominant Ago protein in the heart tissue ^7^; thus, leading to the conclusion that HF‐related miRNAs associated with the Ago1 protein originate from heart tissue and circulate in the bloodstream. Thus, analysing Ago1 serum fraction can overcome this issue.

In the current study, we analysed miRNAs in serum sub‐fractions: exosomes, Ago1‐ and Ago2‐complexes. The exosomal miRNA profile was similar to whole serum miRNA profile identified in the same donor, which is in line with previous publications.^12^, ^21^ Simultaneously, both Ago1‐ and Ago2‐associated miRNAs demonstrated an expression pattern different from those of whole serum, with Ago1 associated miRNAs being highly distinct from serum but also from Ago2‐associated miRNAs. Additionally, we identified an up‐regulation of miR‐222‐3p, miR‐497‐5p and miR‐21‐5p; and down‐regulation of let‐7a‐5p in Ago1 fraction of HF patients compared with those of healthy individuals. No significant differences in expression levels were detected between the two groups of individuals for the same miRNAs in whole serum. Importantly, a combination of these four miRNAs into Ago1‐HF score measured in Ago1 samples resulted in an ROC curve with an AUC of 1. In addition, to that, miR‐22‐3p, miR‐423‐5p, miR‐320a and miR‐92b‐3p that were previously identified as up‐regulated in whole serum of HF patients,^12^ demonstrated better discrimination between healthy individuals and HF patients when assessed in the Ago1 fraction.

We demonstrated increased expression of miR‐21‐5p in the Ago1 complex. In fact, miR‐21‐5p is one of the most widely studied miRNA in the context of cardiovascular diseases and is involved in several pathophysiological mechanisms of heart failure. By activating the ERK‐MAP kinase signalling pathway in cardiac fibroblasts, miR‐21 affects global cardiac structure and function.^31^ In addition, miR‐21 has a protective role in H_2_O_2_‐induced injury of cardiomyocytes.^32^ In agreement with this finding, HF patients with acute decompensation, who had elevated levels of miR‐21 and miR‐126, had better 24‐month survival rate and required less re‐hospitalization than decompensated patients with low levels of these miRNAs.^33^ However, studies comparing miR‐21‐5p expression in HF to healthy individuals are conflicting, demonstrating both up‐regulated and as well as down‐regulated expression.^33^, ^34^, ^35^ The differences may be due to the stage of heart failure, and our finding that miR‐21‐5p expression was elevated in the Ago1 complex, thus suggesting that this miRNA originated from the heart muscle and that a mechanism to reduce heart tissue damage is activated in our HF patients.

Similar to miR‐21‐5p, previous research also confirmed that miR‐222‐3p is linked to cardiovascular diseases. Dubois‐Deruy et al found that miR‐222‐3p is down‐regulated in patients with left ventricular remodelling and it directly interacts with the Mn Superoxide dismutase gene.^36^ In rat fibroblasts, down‐regulation of miR‐221/222 leads to activation of the TGFβ pathway and fibrosis, whereas in HF patients, severe pressure overload dysfunction was associated with lower levels of miR‐222.^37^ In our study, we have found that miR‐222 is higher in HF patients compared with healthy controls, which could contribute to a compensatory mechanism.

We found that let‐7a‐5p expression in the Ago1 fraction was down‐regulated in HF patients compared to that of healthy individuals. Reports on let‐7a expression in cardiovascular disease are conflicting, with down‐regulation of let‐7a in HF or heart injury models ^38^, ^39^ and up‐regulation of let‐7a in the coronary sinus, a blood vessel collecting venous blood from the heart muscle, in HF patients.^40^ Furthermore, in diabetic mice with cardiac hypertrophy and elevated Brain Natriuretic Peptide (BNP), indicating HF, let‐7a was reduced by approximately 80% compared to healthy controls.^39^ let‐7a was also shown to be a crucial part of heart regeneration processes in response to injury in adult zebrafish, a mechanism that does not exist in mammals. Remarkably, this mechanism seems to be dormant in mammals, since down‐regulation of let‐7 and miR‐99/100 using anti‐miRs induces up‐regulation of genes associated with regeneration and reduction of necrotic areas of mouse heart tissue, exposed to hypoxia.^38^ Although heart regeneration does not occur in mammals, we observed down‐regulation of let‐7a‐5p in HF patients, suggesting some attempts at heart regeneration in humans.

Unlike other miRNAs identified in this study, to the best of our knowledge, miR‐497‐5p was not previously associated with any cardiovascular condition. Interestingly, in a recent study by Chen et al miR‐497‐5p was linked to myofibroblast differentiation of lung‐resident mesenchymal stem cells and pulmonary fibrogenesis.^41^ It is important to note that pulmonary fibrogenesis and HF share multiple common features, such as a strong involvement of the TGFβ signalling pathway.^42^, ^43^, ^44^ Indeed, miR‐497‐5p is strongly up‐regulated upon TGFβ stimulation in lung resident mesenchymal stem cells, but also mediates TGFβ induced fibrogenesis, thus forming a positive feedback loop leading to disease deterioration.^41^ Future studies should investigate the role miR‐497‐5p plays in TGFβ ‐mediated heart fibrogenesis.

In conclusion, we identified up‐regulation of miR‐222‐3p, miR‐497‐5p and miR‐21‐5p and down‐regulation of let‐7a‐5p in the Ago1 fraction of HF patients compared with healthy individuals. The differences in expression of these miRNAs could only be observed in Ago1 complexes, but not in whole serum, which is routinely used in most studies. Thus, while Ago1 RNA immunoprecipitation requires a more complicated protocol compared to commonly used serum miRNA identification, analysing Ago1 protein‐associated miRNAs may provide a better glimpse at the failing heart tissue. Provided that Ago1 is the predominant Ago protein in heart tissue and that Ago1‐HF score demonstrates superior discriminating results compared with whole serum HF associated miRNAs, we believe that focusing on Ago1 fraction can help to identify reliable HF biomarkers in future independent studies. Identifying miRNAs that are associated with HF pathology can potentially provide diagnostic and prognostic tools, and further our understanding of the molecular mechanisms of a heart failure. Future studies should address the effects of miRNAs, or their inhibitors, and their potential as a therapeutic tool to treat HF.

## CONFLICT OF INTEREST

This work was supported by Rosetta Genomics Ltd, Rehovot, Israel. The following authors are employed by Rosetta: Eti Meiri, Nir Dromi, Sharon Kredo‐Russo, Hila Benjamin, Sarit Tabak, Hagai Marmor, Maria Motin, Danit Lebanony, Gila Lithwick‐Yanai, and Etti Kadosh.

## AUTHOR CONTRIBUTION

EM designed the study, performed the research and contributed to the manuscript writing. NV performed data analysis, statistics and wrote the manuscript. HM and SKR participated in the study design and performed the research. ST, DL, MM, EK performed the research. ND and HB performed data analysis and statistics. GLY performed data analysis, statistics and contributed to drafting the manuscript. CK supplied the monoclonal anti‐Ago antibody and protocols for RIP. LGR contributed to the manuscript writing. OA designed the study, recruited study participants and contributed to the manuscript writing. All authors have read and approved the final manuscript.

## Supporting information

Fig S1

Table S1

Supplementary Material

## Data Availability

The data that support the findings of this study are available on request from the corresponding author. The data are not publicly available due to privacy or ethical restrictions.
