# Supplementary figures and images for: Differential expression of microRNA in serum fractions and association of Argonaute 1 microRNAs with heart failure
Source: J Cell Mol Med. 2020 May 13;24(12):6586–95. doi: 10.1111/jcmm.15306 (PMC7299714; doi:10.1111/jcmm.15306)

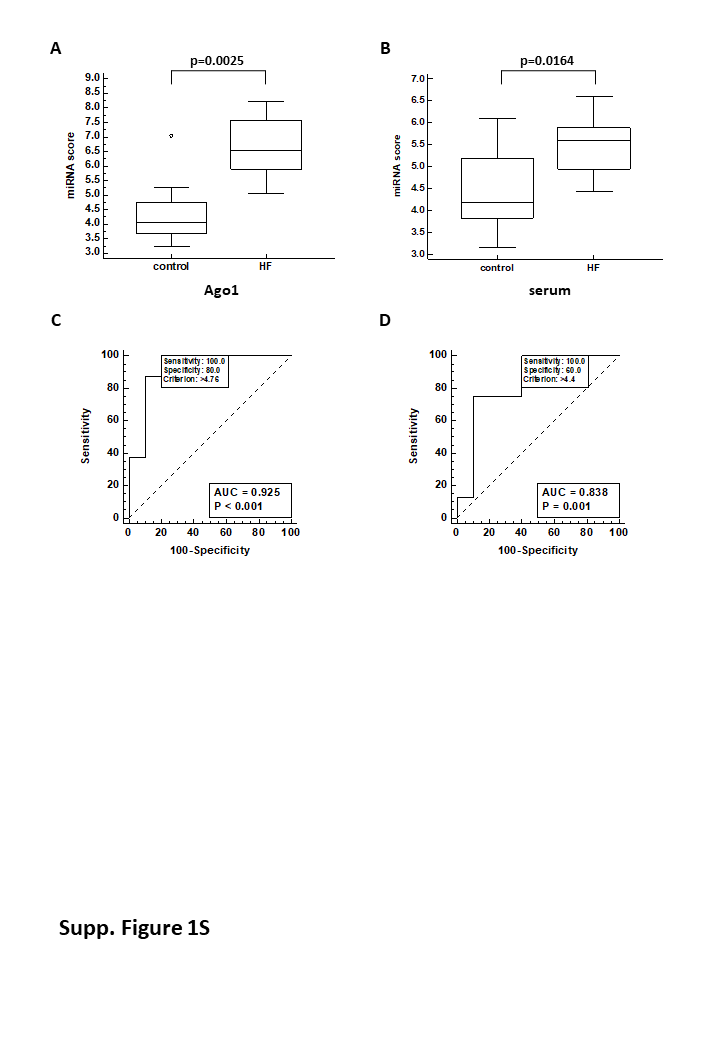

Supplement: Supplementary file 1 — Fig S1 [file JCMM-24-6586-s001.tif]
