# Supplementary material for: Differential expression of microRNA in serum fractions and association of Argonaute 1 microRNAs with heart failure
Source: J Cell Mol Med. 2020 May 13;24(12):6586–95. doi: 10.1111/jcmm.15306 (PMC7299714; doi:10.1111/jcmm.15306)
Supplement: Supplementary file 3 — Supplementary Material [file JCMM-24-6586-s003.docx]

**Supp. Figure 1S:**

(A,B) Values of HF score, calculated as a sum of miR-22-3p, miR-423-5p, miR-320a and miR-92b-3p relative expression values, in Ago1 RIP (A) and whole serum (B). *p* values were calculated using the Mann-Whitney test. (C,D) ROC curve for HF score of healthy individuals versus HF patients in Ago1 RIP (C) and whole serum (D).

**Supp. Table S1:**

Relative expression of 47 pre-selected miRNAs of HF patients (n=8) compared with healthy individuals (n=10) in Ago RIP and whole serum. *p* value was calculated using the Mann-Whitney test.
