# Supplementary material for: Differential expression of microRNA in serum fractions and association of Argonaute 1 microRNAs with heart failure
Source: J Cell Mol Med. 2020 May 13;24(12):6586–95. doi: 10.1111/jcmm.15306 (PMC7299714; doi:10.1111/jcmm.15306)
Supplement: Supplementary file 3 [file JCMM-24-6586-s002.docx]

	Ago		serum	
miR_name	Fold change	p value	Fold change	p value
hsa-let-7a-5p	0.56	0.013	0.62	0.091
hsa-let-7c-5p	0.81	0.248	1.39	0.424
hsa-let-7f-5p	0.62	0.110	0.64	0.286
hsa-miR-122-5p	0.70	0.328	0.73	0.689
hsa-miR-126-3p	0.83	0.010	0.67	0.003
hsa-miR-126-5p	0.75	0.110	0.50	0.051
hsa-miR-130a-3p	0.90	0.477	1.01	0.929
hsa-miR-133a-3p	1.00	1.000	1.00	0.274
hsa-miR-142-5p	1.03	0.929	0.89	0.424
hsa-miR-143-3p	0.60	0.183	1.25	0.534
hsa-miR-144-3p	1.00	0.515	1.36	0.689
hsa-miR-145-5p	0.91	0.689	0.87	0.286
hsa-miR-146a-5p	0.85	0.929	0.72	0.286
hsa-miR-146b-5p	0.88	0.722	0.71	0.248
hsa-miR-148a-3p	1.00	0.859	0.81	0.756
hsa-miR-150-5p	0.63	0.214	0.70	0.183
hsa-miR-15a-5p	0.75	0.041	1.05	0.477
hsa-miR-15b-5p	0.89	0.564	0.95	0.929
hsa-miR-16-5p	0.96	0.657	1.42	0.100
hsa-miR-17-5p	0.73	0.069	1.01	0.722
hsa-miR-181a-5p	0.90	0.248	0.80	0.198
hsa-miR-194-5p	1.33	0.374	1.10	1.000
hsa-miR-195-5p	0.52	0.083	0.74	0.424
hsa-miR-199b-5p	0.62	0.062	0.28	0.050
hsa-miR-210-3p	1.30	0.594	1.69	0.008
hsa-miR-21-5p	1.63	0.021	1.47	0.091
hsa-miR-221-3p	0.95	0.307	0.54	0.069
hsa-miR-222-3p	1.75	0.011	1.43	0.100
hsa-miR-223-3p	0.87	0.534	0.57	0.062
hsa-miR-22-3p	1.78	0.033	1.76	0.051
hsa-miR-23a-3p	0.99	0.859	0.87	0.068
hsa-miR-27a-3p	0.95	0.477	0.73	0.015
hsa-miR-27b-3p	0.82	0.021	0.58	0.001
hsa-miR-29a-3p	1.10	0.286	1.12	0.424
hsa-miR-29c-3p	0.94	0.859	1.48	0.091
hsa-miR-30a-5p	1.43	0.010	1.20	0.214
hsa-miR-320a	1.44	0.003	1.63	0.008
hsa-miR-324-5p	0.40	0.154	0.62	0.068
hsa-miR-342-3p	0.89	0.374	0.71	0.214
hsa-miR-375	1.04	0.756	0.88	0.965
hsa-miR-378a-3p	0.93	0.594	1.26	0.424
hsa-miR-423-5p	1.44	0.024	1.57	0.002
hsa-miR-425-5p	1.14	0.328	1.71	0.026
hsa-miR-451a	0.97	0.625	1.17	0.689
hsa-miR-497-5p	1.76	0.016	0.86	0.722
hsa-miR-551b-3p	0.02	0.320	1.00	0.274
hsa-miR-92b-3p	0.87	0.328	1.77	0.008
